# Multiplex Quantitative Analysis of Tumor-Infiltrating Lymphocytes, Cancer-Associated Fibroblasts, and CD200 in Pancreatic Cancer

**DOI:** 10.3390/cancers13215501

**Published:** 2021-11-02

**Authors:** Tyler MacNeil, Ioannis A. Vathiotis, Saba Shafi, Thazin Nwe Aung, Jon Zugazagoitia, Aaron M. Gruver, Kyla Driscoll, David L. Rimm

**Affiliations:** 1 Department of Pathology, Yale School of Medicine, New Haven, CT 06510, USA; tyler.macneil@yale.edu (T.M.); ioannis.vathiotis@yale.edu (I.A.V.); saba.shafi@yale.edu (S.S.); thazin.aung@yale.edu (T.N.A.); jon.zugazagoitia@yale.edu (J.Z.); 2 Eli Lilly and Company, Indianapolis, IN 46285, USA; gruver_aaron_m@lilly.com (A.M.G.); driscoll_kyla@lilly.com (K.D.)

**Keywords:** PDAC, immunotherapy, lymphocytes, fibroblasts, CD200

## Abstract

**Simple Summary:**

Pancreatic cancer is one of the most lethal types of cancer, and immunotherapy treatment options for these patients are limited by the characteristically “cold” tumor microenvironment. In this work, we analyze the expression levels and prognostic value of stromal tumor-infiltrating lymphocyte (CD4, CD8, and CD20) and cancer-associated fibroblast (Thy-1, FAP, and SMA) subpopulations in a cohort of pancreatic ductal adenocarcinoma patients. We additionally characterize the expression and prognostic value of CD200, a potential target for immune checkpoint blockade in these patients. CD8 and FAP were found to have prognostic significance for progression-free survival and overall survival after multivariate analysis. CD200 expression was heterogeneous in tumor and stromal cells and did not demonstrate prognostic value in this cohort. Our results point to CD8 and FAP as potential prognostic biomarkers and demonstrate the heterogeneous expression pattern of CD200 in patients with pancreatic ductal adenocarcinoma.

**Abstract:**

Pancreatic cancer is marked by a desmoplastic tumor microenvironment and low tumor immunogenicity, making it difficult for immunotherapy drugs to improve outcomes for patients. Tumor-infiltrating lymphocytes (TILs) and cancer-associated fibroblasts (CAFs) are seen in the tumor microenvironment of patients with pancreatic ductal adenocarcinoma (PDAC). In this work, we sought to characterize the expression levels and potential prognostic value of TILs (CD4, CD8, and CD20) and CAFs (Thy-1, FAP, and SMA) in a large retrospective cohort of PDAC patients. Additionally, we investigated the expression levels and prognostic significance of CD200, an immunoinhibitory protein that has shown interest as a potential target for immune checkpoint blockade. We measured the expression levels of these seven proteins with multiplexed immunofluorescence staining and quantitative immunofluorescence (QIF). We found CD8 and FAP to be independent predictors of progression-free survival and overall survival. CD200 was found to be heterogeneously expressed in both the tumor and stromal compartments of PDAC, with the majority of patients having positive stromal expression and negative tumor expression. This work demonstrates the potential clinical utility of CD8 and FAP in PDAC patients, and it sheds light on the expression patterns of CD200 in pancreatic cancer as the protein is being tested as a target for immune checkpoint blockade.

## 1. Introduction

Pancreatic cancer is one of the most lethal types of cancer in the United States. In 2021, pancreatic cancer is listed as the third leading cause of cancer deaths and as having the lowest survival rate of any cancer, with only a 10% five-year survival rate across all stages [1]. Survival rates have increased for patients with low-stage pancreatic cancer with the use of surveillance programs and genetic screening [2]; however, patients with locally advanced or metastatic disease have limited options for treatment. About 20% of pancreatic cancer patients are eligible for surgery, and other treatment options include chemotherapy, radiotherapy, and immunotherapy, the latter of which is only approved for a small portion of patients with specific genetic profiles. Immune therapies have been tested in patients with pancreatic cancer in both the neoadjuvant and adjuvant as well as metastatic setting but have shown limited positive results [3,4,5,6,7]. A significant hurdle in finding success with immune checkpoint inhibitors in pancreatic ductal adenocarcinoma (PDAC) is the characteristic desmoplastic stromal region and inherent lack of immunogenicity of these tumors [8,9,10].

The tumor microenvironment includes both tumor and immune cells and is the site of action for many immunotherapy drugs. Cells that commonly inhabit these areas often fall under the categories of tumor-infiltrating lymphocytes (TILs) and cancer-associated fibroblasts (CAFs). Microenvironmental biomarkers such as CD8 and FAP have shown clinical utility in a variety of cancers, including melanoma and breast cancer [11,12,13,14]. In the PDAC tumor microenvironment, immunosuppressive cells such as myeloid-derived suppressor cells (MDSCs) contribute to the lack of immunogenicity in PDAC stromal areas [15,16]. Various groups have attempted to characterize the stromal region of pancreatic cancer, including genetically [17] and by the expression of collagen [18]. Further investigation of proteins in the stroma in PDAC is needed to accurately define the region and to find novel biomarkers and new routes for immunotherapy and other treatment modalities in pancreatic cancer.

CD200 is a membrane-bound, type I glycoprotein, and it binds with its receptor to inhibit the immune system. The protein is expressed by B cells, T cells, endothelial cells, and other cell types. CD200 is highly expressed in neuroendocrine tissues, and its expression was found by our group to be heterogeneous in the tumor and stromal compartments of lung cancer patients [19,20]. The ligand has demonstrated clinical implications in a variety of solid and liquid tumors. CD200 expression has shown diagnostic capabilities in lymphoid malignancies and prognostic significance in acute myeloid leukemia, and the protein has been investigated for utility as a target for immune checkpoint blockade [21,22,23,24,25].

In the present study, we utilize multiplexed quantitative immunofluorescence (QIF) to measure the expression of TILs (through CD4-, CD8-, and CD20-positive cells) and CAFs (through Thy-1-, FAP-, and SMA-positive cells) in the PDAC stroma. Additionally, we measure the expression of CD200 in both the tumor and stromal compartments of patients in the same cohort. Finally, we assess the prognostic value of the stromal proteins and CD200 in the cohort.

## 2. Materials and Methods

### 2.1. Patient Cohorts and TMA Construction

We stained and analyzed retrospectively collected, formalin-fixed, paraffin-embedded (FFPE) tumor specimens contained in a tissue microarray (TMA) format. The specimens were collected and used with specific consent or waiver of consent under approval from the Yale Human Investigation Committee protocol #9505008219.

The PDAC cohort we analyzed (YTMA454) contained 238 tumor specimens resected between 2010 and 2017. Two blocks, each with one core per patient, of YTMA454 were used for our analysis. Clinicopathologic data were collected from patients’ medical record files, and Table 1 summarizes the characteristics of patients in the cohort. No special information was collected on treatment. The number of quantified tumor specimens differs from the total number of tumors in the cohort due to the loss of spots after TMA construction or removal of histospots with tissue folding or other artifacts seen on inspection while quantifying them.

We also built a custom “index” TMA (YTMA434) for reagent titration, assay validation, and reproducibility assessments for CD200. This index TMA contained cores of normal and cancerous pancreas tissue, as well as FFPE-prepared parental A20 cells and A20 cells transfected to overexpress CD200. TMA construction with cell lines has been published in detail elsewhere [26].

### 2.2. Multiplexed Immunofluorescence Staining Protocol

Freshly cut TMA slides were subjected to deparaffinization and antigen retrieval with ethylenediaminetetraacetic acid buffer (pH = 8.0) at 97 °C for 20 min using a pressure boiling container (PT Module; Thermo Fisher Scientific, Waltham, MA, USA). Slides were then incubated in 0.3% hydrogen peroxide in methanol for 30 min at room temperature and then for 30 additional minutes in 0.3% bovine serum albumin with 0.05% Tween-20 blocking solution at room temperature. The TMA slides were incubated with cocktails of primary monoclonal antibodies against CD4 (1:100; SP35; Spring Bioscience, Pleasanton, CA, USA), CD8 (1:250; C8/144B; Agilent, Santa Clara, CA, USA), and CD20 (1:150; L26; Agilent, Santa Clara, CA, USA) for TIL quantification, or against Thy-1 (1:10,000; 7E1B11; Abcam, Cambridge, MA, USA), FAP (1:500; EPR20021; Abcam, Cambridge, MA, USA), and α-SMA (1:500; 1A4; Agilent, Santa Clara, CA, USA) for CAF quantification, for 1 h at room temperature. The TMA cuts were incubated sequentially with two horseradish peroxidase (HRP)–conjugated secondary antibodies for 1 h at room temperature, followed by tyramide-based HRP activation for 10 min and then with 1 mmol/L benzoic hydrazide with 0.15% hydrogen peroxide twice, for 7 min each to quench HRP activation. The secondary antibodies used were rabbit EnVision amplification reagent (Agilent, Santa Clara, CA, USA) and anti-mouse IgG1 (1:100; eBioscience, San Diego, CA, USA), and the HRP activating substrates were biotinylated tyramide (1:50; Akoya Biosciences, Marlborough, MA, USA) and TSA Plus Cy3 Tyramide (1:100; Akoya Biosciences, Marlborough, MA, USA), respectively. The slides were then incubated with a final secondary antibody against mouse IgG2a (1:200; Abcam, Cambridge, MA, USA) for 1 h at room temperature and a final HRP activating substrate, Cy5 tyramide (1:50; Akoya Biosciences, Marlborough, MA, USA), for 10 min at room temperature, before being incubated with Alexa750-Streptavidin (1:100; Invitrogen, Carlsbad, CA, USA) for 1 h at room temperature. The TMA cuts were then incubated with a monoclonal mouse anti-cytokeratin antibody (1:100; AE1/AE3; Agilent, Santa Clara, CA, USA) for one hour at room temperature and with Alexa488-conjugated goat anti-mouse (1:100; Invitrogen, Carlsbad, CA, USA) for one hour at room temperature to visualize cytokeratin-positive cells. Finally, the slides were incubated for 20 min with 4,6-diamidino-2-phenylindole (DAPI) diluted at 1:1000 and mounted with ProLong mounting medium (ProLong Gold; Invitrogen, Carlsbad, CA, USA).

For experiments with CD200, fresh TMA cuts were deparaffinized, and antigen retrieval was completed with ethylenediaminetetraacetic acid buffer (pH = 9.0) at 97 °C for 20 min. After completing the blocking protocol described above, slides were incubated in a cocktail of primary antibodies against CD200 (5F3D6; Proteintech, Rosemont, IL, USA), at the optimal concentration of 1 µg/mL, and against cytokeratin with a polyclonal rabbit anti-cytokeratin antibody (1:100; Agilent, Santa Clara, CA, USA) for 1.5 h at room temperature. Slides were incubated for one hour at room temperature with Alexa546-conjugated goat anti-rabbit secondary antibody (Invitrogen, Carlsbad, CA, USA) diluted 1:100 in rabbit EnVision. The slides were then incubated for 10 min at room temperature with Cy5 diluted 1:50, incubated for 20 min with DAPI diluted 1:500, and finally mounted in ProLong medium as described above. Control slides from the CD200 index array (YTMA434) were used in each experiment to ensure assay reproducibility. All antibodies we used to quantify the proteins of interest were optimized and validated by our group prior to use in this cohort [19,27].

### 2.3. Fluorescence Signal Quantification

To quantify the expression of the TIL, CAF, and CD200 proteins, we used automated QIF analysis with the AQUA platform, as previously described [28]. To generate QIF scores in the tumor compartment, the sums of pixel intensities for our targets were divided by the area of cytokeratin positivity, which resulted in continuous scores directly proportional to the target concentrations. Similarly, QIF scores for the proteins expressed within the stromal compartment were generated by dividing the sum of the pixel intensities by the area of the DAPI compartment minus the tumor compartment. QIF scores were normalized for exposure time and bit depth for comparison in analysis. Histospots were reviewed, and spots with staining artifacts or those that were less than 2% of the tumor area (cytokeratin staining) were excluded from the analysis. For all biomarkers, scores were averaged from two independent TMA blocks of YTMA454. The blocks each contained an individual tumor core taken from a nonadjacent location for each patient. CD200 positivity in the tumor and stroma in YTMA454 was determined visually by reviewing each histospot for CD200 expression in each compartment. The median of scores for all proteins and the thresholds for visual positivity of CD200 within each compartment were used as the cutpoints for survival analysis.

### 2.4. Statistical Analysis

The Pearson correlation coefficient was used to assess the linear association between two continuous variables. Survival curves were created with the Kaplan–Meier product-limit method, and log-rank tests were used to compare them. Multivariate Cox proportional hazards models were conducted and included age, stage, and neoadjuvant chemotherapy status as covariates [29,30,31,32]. Statistical analysis was performed using the GraphPad Prism 8.0 software (GraphPad Software, San Diego, CA, USA) and JMP Pro 15 software (SAS Institute, Cary, NC, USA). Hypothesis testing was conducted at a two-sided significance level, with α equal to 0.05.

## 3. Results

### 3.1. Expression of Stromal Proteins and CD200 in Pancreatic Cancer

**Figure 1:**
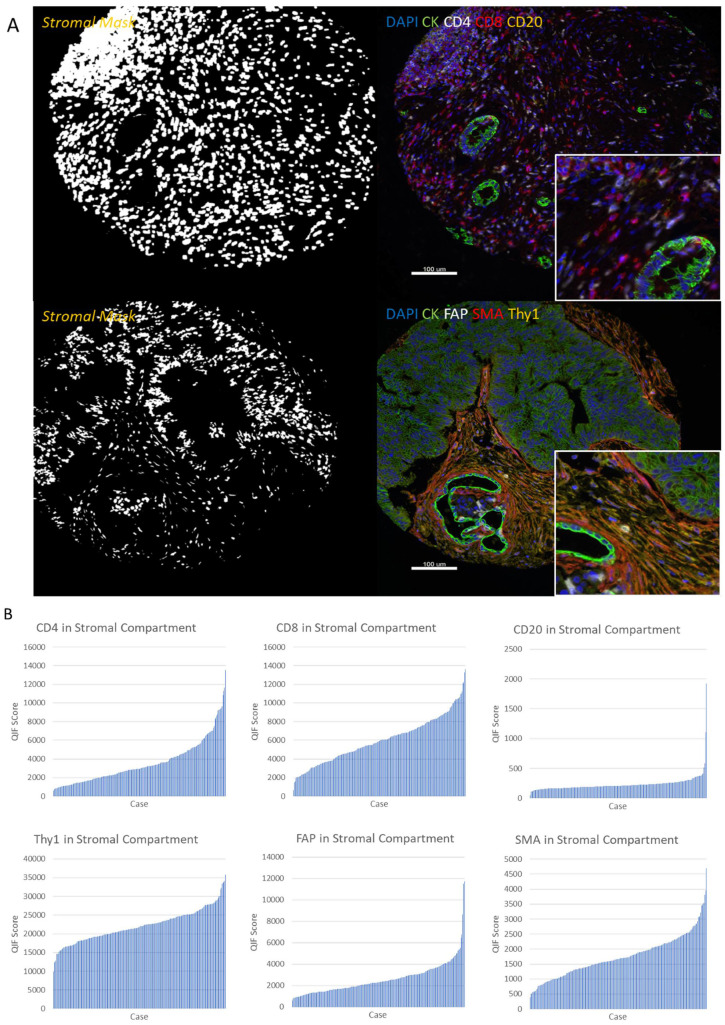

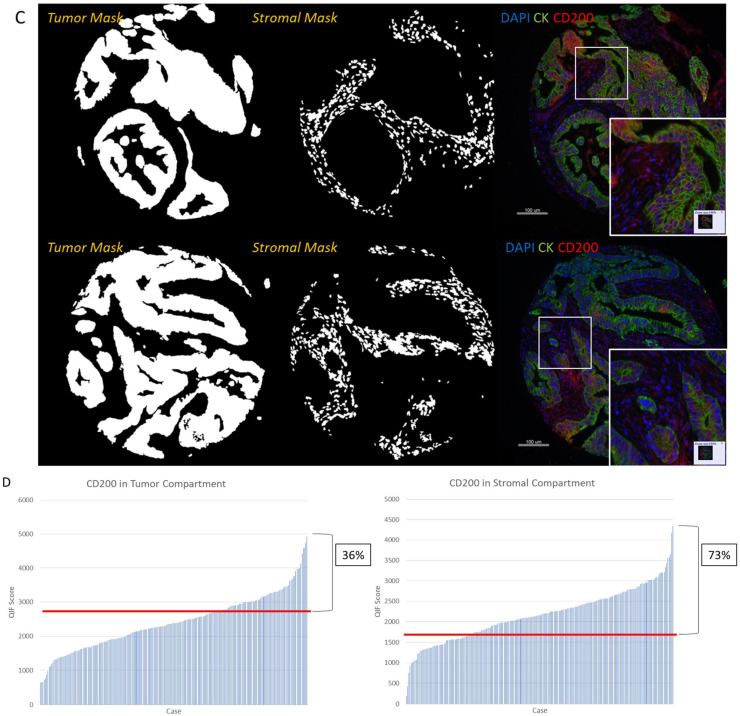
Expression patterns of TILs, CAFs, and CD200 in PDAC patients. (**A**) TIL markers (CD4, CD8, and CD20) were characterized utilizing multiplexed fluorescence staining, with CD4 represented in white, CD8 in red, and CD20 in yellow. CAF markers (Thy-1, FAP, and SMA) were characterized with the same staining procedure, where FAP is represented in white, SMA is shown in red, and Thy-1 is in yellow. Representative images of stromal masks are depicted as well. (**B**) Dynamic range charts for TIL and CAF proteins in the stromal compartment of tumors in YTMA454. (**C**) CD200 was stained with a multiplex fluorescence staining protocol as well and is represented in red, alongside representative images of tumor and stromal masks. (**D**) Dynamic range of CD200 in both the tumor and stromal compartments of patients, with red lines depicting the visually determined thresholds for assay limit of detection. Abbreviations: DAPI, 4,6-diamidino-2-phenylindole; CK, cytokeratin; QIF, quantitative immunofluorescence.

TIL and CAF proteins were measured in the stromal compartment in the YTMA454 cohort (Figure 1A). Quantified TIL and CAF marker expression showed variable expression patterns across patients (Figure 1B). CD4 had the broadest dynamic range of expression, while CD20 was expressed at a relatively low level across the cohort of patients. In the stroma, the protein with the smallest dynamic range of expression was Thy-1, since this protein is expressed broadly by all CAF cells. Regressions of pairs of histospots were assessed for TIL and CAF markers to test for protein expression heterogeneity (Figure S1). CD8, Thy-1, and FAP expression levels were relatively well correlated between different blocks of YTMA454, with R^2^ values equal to 0.40, 0.38, and 0.39, respectively. CD4 and SMA demonstrated greater heterogeneity of expression across blocks, with R^2^ values of 0.22 for each.

CD200 demonstrated visibly heterogeneous expression patterns in both tumor and stromal cells, as observed in two representative tumor cores (Figure 1C). CD200 was quantified in the tumor and stromal compartments (Figure 1D). The protein was “positive” in the tumor compartment in 36% of patients, where positive is defined by the visually determined limit of detection (cutpoint) of the assay. CD200 was positive in the stromal compartment in 73% of patients in the YTMA454 cohort. In tumor cells, CD200 demonstrated low correlations of expression between different blocks in all positive patients (R^2^ = 0.16; Figure S2A,B). Likewise, the patients’ tumor specimens were compared by CD200 stromal compartment scores across blocks of YTMA454 (Figure S2C,D). In the stromal cells, low R^2^ values for all patients and for patients separated by signal or noise expression scores show that there is high heterogeneity of CD200 stromal expression across PDAC tumors. The R^2^ value for CD200 expression in the tumor versus stromal compartment of patients was 0.63 (Figure S3A). This value demonstrates that across PDAC patients, patients who have high CD200 tumor expression tend to also have high CD200 stromal expression. We found a similar relationship of CD200 compartmental expression in lung cancer patients as well [19].

Next, TIL and CAF proteins’ expression values in the stroma were compared to CD200 stromal expression values (Figure S3B). CD4 and CD8 had R^2^ values of 0.15 and 0.25, respectively, when compared with CD200 expression, while other TIL and CAF markers showed very low regression values.

### 3.2. Prognostic Significance of TIL and CAF Proteins and CD200 in Pancreatic Cancer

**Figure 2:**
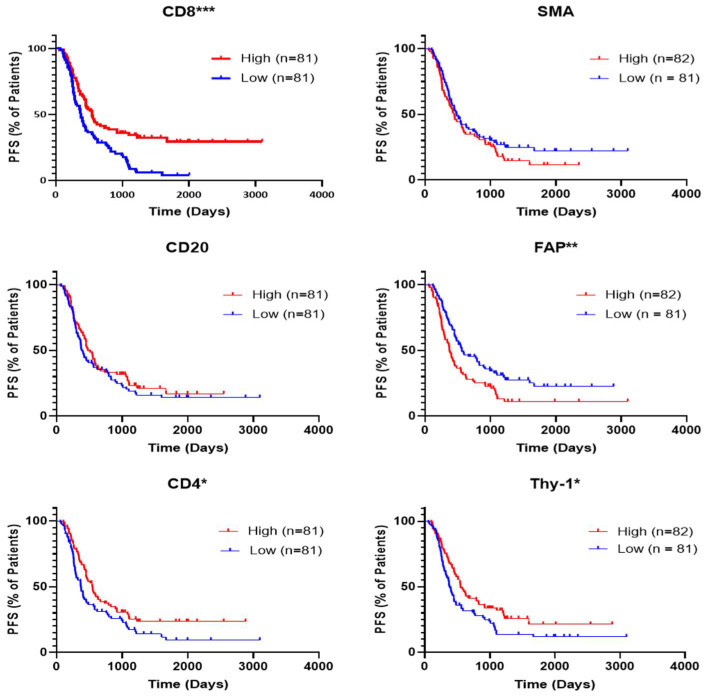
Progression-free survival of patients in YTMA454 based on expression of TIL and CAF biomarkers. Representative Kaplan–Meier curves show the progression-free survival of different groups of patients in YTMA454. The median values of expression for each marker were used as the cutpoints to define high- and low-expressing patients. Abbreviations: PFS, progression-free survival; * *p* < 0.05; ** *p* < 0.01; *** *p* < 0.0001.

Progression-free survival (PFS) was tested for patients of YTMA454 based on median stromal expression of TIL and CAF biomarkers (Figure 2). Statistically significant prolonged PFS was observed for higher expression of CD4, CD8, and Thy-1 and for lower expression of FAP. Trends can be observed for better PFS in patients with higher CD20 expression and lower SMA expression. Multivariate Cox proportional hazards model analysis was conducted using age, tumor stage, and neoadjuvant chemotherapy status as covariates to test the hazard ratio (HR; high expression over low expression) and 95% confidence interval (CI) for each protein of interest (Table 2). Multivariate analysis showed only CD8 (HR = 0.52, CI = 0.34–0.80; *p* = 0.0028) and FAP (HR = 1.49, CI = 1.03–2.17; *p* = 0.037) remained significant for predicting PFS. High CD8 and low FAP were found to significantly predict improved patient overall survival (OS), and multivariate analysis showed these two markers remained statistically significant (Figure S4). After multivariate analysis with these two proteins together with age, stage, and neoadjuvant chemotherapy status, both CD8 (HR = 0.64, CI = 0.42–0.97; *p* = 0.035) and FAP (HR = 1.63, CI = 1.07–2.46; *p* = 0.022) were found to predict OS in the patients of YTMA454 (Table S1).

**Figure 3:**
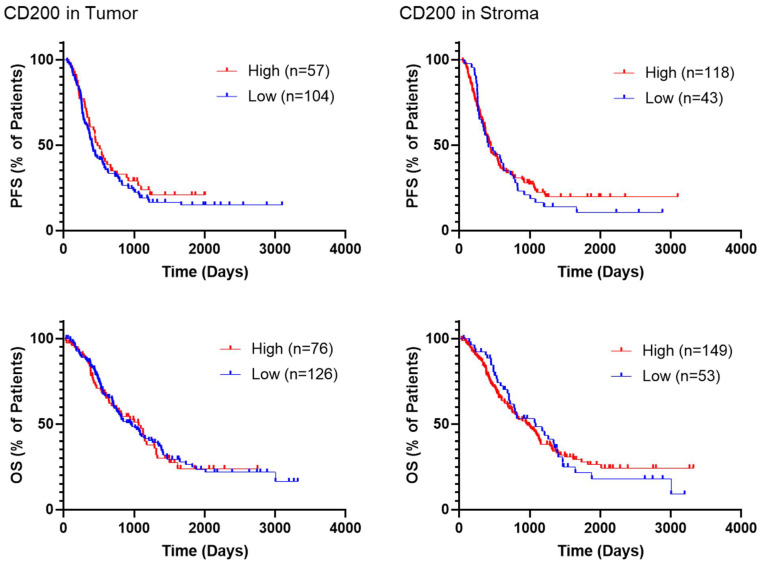
Survival of patients in YTMA454 based on CD200 expression. Representative Kaplan–Meier curves demonstrate the survival of different groups of patients in YTMA454. The visually determined thresholds of positivity were used as cutpoints to define high- and low-expressing patients for CD200 in both the tumor and stromal compartments. Abbreviations: PFS, progression-free survival; OS, overall survival.

PFS and OS were analyzed based on CD200 expression in YTMA454 (Figure 3) using the visual limit of detection (Figure 1D). There were no statistically significant associations observed for PFS or for OS based on CD200 visual expression, including after multivariate analyses (Table 3). A similar lack of prognostic significance was found when CD200 expression was cut at the median QIF score (Figure S5).

## 4. Discussion

In this study, we quantitatively analyzed the expression of TIL and CAF proteins in the stroma of patients with PDAC. We also characterized the expression of CD200 in both the tumor and stromal compartments of PDAC patients. We found TIL and CAF markers to be variably expressed across this cohort of PDAC patients, and we found CD200 to be heterogeneously expressed in both the tumor and stromal compartments of the cohort, with 36% of patients demonstrating positive tumor expression of the protein and 73% of patients showing positive stromal expression. We analyzed the prognostic significance of these proteins, and CD8 and FAP remained statistically significant after multivariate analyses, demonstrating these two proteins are independent predictors of PFS and OS in PDAC. CD200 was not found to predict patient outcome in this cohort.

CD8 was found to be an independent predictor of PFS and OS in this cohort of PDAC patients. Although prognosis was predicted by other TIL markers, CD8 was the only protein to remain statistically significant after multivariate analysis. CD8 was also one of the stromal proteins to demonstrate relatively homogeneous expression patterns across different tumor cores in patients. In pancreatic cancer, CD8-positive cytotoxic T cells are associated with better prognosis [33,34]. A variety of therapies have been tested to increase CD8 T cell infiltration into the stroma of PDAC, including chimeric antigen receptor T cell therapies, CD40 antibody blockade, and CCL2/CCR2 blockade [35,36,37,38]. Additionally, depletion of immunosuppressive cells in the tumor microenvironment of PDAC including T regulatory cells, tumor-associated dendritic cells, and MDSCs have been shown to increase CD8 cell infiltration [39,40]. Beyond pancreatic cancer, CD8 is a prognostic factor in other cancers including breast cancer and melanoma [12,41]. The immunosuppressive effects of PD-1 and PD-L1 and their associations with CD8 cells in pancreatic cancer suggest the potential usefulness of CD8 as a biomarker for immunotherapies in PDAC [42,43,44].

FAP was also found to be an independent predictor of both PFS and OS in pancreatic cancer patients. Although Thy-1 had some prognostic value as well, FAP alone remained significant after multivariate analyses. FAP and Thy-1 also showed consistent expression patterns across different blocks of YMA454. FAP is a cell-surface protease, and the protein was named for its prevalence on reactive fibroblast cells, particularly in different types of cancer [45]. Its effects in cancers are through both enzymatic and non-enzymatic means. The expression of FAP has been found to promote immunosuppression and worse outcomes for patients with several types of cancers. Targeting FAP has been a topic of interest in oncology treatment, and in pancreatic cancer, blocking of FAP has led to better outcomes and also to increased infiltration of CD8 T cells [46,47,48,49]. In particular, Fabre et al. recently used a monoclonal antibody directly targeting FAP in a preclinical mouse model and showed blocking of the protein to lead to better outcomes and higher CD8 infiltration in PDAC. FAP-positive CAF cells have additionally been investigated for their crosstalk with cytotoxic natural killer cells in PDAC [50]. The finding of FAP as an independent prognostic factor for both PFS and OS in PDAC demonstrates the likely immunosuppressive effects the protein has in the pancreatic cancer tumor microenvironment as well as the potential for finding treatment strategies involving it.

CD200 was found to be heterogeneously expressed in PDAC. The protein was found to have low regressions in the tumor and stromal compartments between blocks of YTMA454, a high correlation between its expression in the tumor versus stromal compartment, and modest correlations of expression with CD4 and CD8 in patients relative to other TIL and CAF markers. In a study by Choueiry et al., CD200 expression in the stroma was found to promote immunosuppression as a potential regulator of MDSCs, and blocking the protein improved the efficacy of PD-1 immune checkpoint blockade [15]. Our group found CD200 to be heterogeneously expressed in lung cancer and to have correlated expression in the tumor and stromal compartments [19]. Although we found no prognostic significance of CD200 in this cohort of pancreatic cancer patients, this does not rule out the potential predictive value of this marker, as the protein is being investigated for immune checkpoint blockade drugs. The ligand has garnered interest as a target for checkpoint blockade, and specifically, the drug samalizumab showed promising results in a phase I clinical trial in chronic lymphocytic leukemia patients [25]. Finally, our findings of moderate correlations between CD200 and CD4 and CD8 indicate potential relationships between CD200 and T cells, or the potential of CD200 expression on T cells, in PDAC.

There are several limitations to our study. Although the exact type of surgery was not collected from patients’ medical records when the cohort was created, all patients got treated with curative intent. Additionally, most of the patients (79%) received postoperative chemotherapy, while only a small fraction received postoperative radiotherapy (13%), according to local standards of care. Considering the fact that our cohort represents a “real-world” collection, we believe that staging information might provide an adequate surrogate for the standard of care of patients’ treatment. Additionally, we used two independent TMA blocks to quantify patient tumors. This means that for each patient, two cores were scored and averaged to result in one score. Whole-tissue slides are the standard in the clinical setting and represent 200 times more tissue area but make high-throughput quantification more challenging. We used the median and visual expression threshold as the cutpoints for positivity for our biomarkers of interest. Although statistically significant results were found with these cutpoints, for clinical utility of these biomarkers, validation cohorts of PDAC patients could be used to identify the optimal cutpoints for expression of the proteins.

Overall, we quantitatively measured the expression of TIL and CAF proteins and CD200 in PDAC. The desmoplastic stroma in pancreatic cancer leads to a lack of immunogenicity and poor efficacy of immunotherapy drugs. In this study, we identified higher CD8 expression and lower FAP expression in the stroma to be independently predictive of prognosis in a cohort of PDAC patients. We additionally found CD200, an immunosuppressive protein and candidate for immune checkpoint blockade, to be heterogeneously expressed in PDAC. Our results demonstrate the importance of finding methods to increase the infiltration of CD8 T cells in pancreatic cancer. Further, blocking FAP might improve outcomes in PDAC patients and possibly increase CD8 cell levels. Lastly, CD200 was expressed in the stroma in the majority of these PDAC patients, and blockade of this protein could be another potential route for treating pancreatic cancer patients.

## 5. Conclusions

In conclusion, we quantitatively analyzed the expression levels of TIL and CAF stromal proteins and CD200, an immunoinhibitory protein, in a cohort of PDAC patients. We identified CD8 and FAP to independently predict PFS and OS in this cohort. CD200 expression was found to be heterogeneously expressed in the stroma in the majority of patients and in tumor cells in the minority of patients. Our results demonstrate the potential utility of activating CD8 cell infiltration in the tumor microenvironment of PDAC, inhibiting FAP and its mechanisms, and blockade of CD200, as well as the potential of each of these proteins in companion diagnostics tests for immunotherapy treatments.

## Figures and Tables

**Table 1:** Clinicopathologic characteristics of patients contained in the YTMA454 cohort.

		***n* (%)**
Age	Median (Range)	69 (34–87)
Sex	Male	116 (50)
	Female	116 (50)
Stage	I	15 (6.5)
	II	199 (85.8)
	III	3 (1.3)
	IV	5 (2.2)
Tobacco	Former	110 (47.4)
	Current	24 (10.3)
	Never	87 (37.5)
Alcohol	Yes	94 (40.5)
	No	121 (52.2)
Neoadjuvant chemotherapy	Yes	121 (52.2)
	No	111 (47.8)
	Former	5 (2.2)
Death	Yes	145 (62.5)
	No	81 (34.9)
OS (Days)	Median (IQR)	756 (923)
Recurrence	Yes	137 (59.1)
	No	39 (16.8)
PFS (Days)	Median (IQR)	448 (707)

**Table 2:** Univariate and multivariate analyses of progression-free survival of patients in YTMA454 based on expression of TIL and CAF markers. Abbreviations: HR, hazard ratio; CI, confidence interval.

Protein	Univariate Analysis		Multivariate Analysis per Variable		Multivariate Analysis CD4, CD8, Thy-1, and FAP	
High/Low	HR (95% CI)	*p* Value	HR (95% CI)	*p* Value	HR (95% CI)	*p* Value
CD4	0.68 (0.48–0.96)	**0.027**	0.66 (0.46–0.96)	**0.027**	0.95 (0.63–1.44)	0.81
CD8	0.54 (0.38–0.76)	**0.0003**	0.47 (0.32–0.68)	**<0.0001**	0.52 (0.34–0.80)	**0.0028**
CD20	0.84 (0.59–1.18)	0.31	0.81 (0.57–1.16)	0.26		
Thy-1	0.66 (0.47–0.94)	**0.02**	0.65 (0.45–0.95)	**0.025**	0.70 (0.47–1.03)	0.69
FAP	1.66 (1.17–2.36)	**0.0039**	1.62 (1.13–2.33)	**0.0088**	1.49 (1.03–2.17)	**0.037**
SMA	1.25 (0.88–1.77)	0.21	1.27 (0.89–1.82)	0.19		

**Table 3:** Univariate and multivariate analyses of patient survival in YTMA454 based on visually determined expression of CD200 in the tumor and stromal compartments. Abbreviations: PFS, progression-free survival; OS, overall survival; HR, hazard ratio; CI, confidence interval.

Function	Protein	Univariate Analysis		Multivariate Analysis per Variable	
	High/Low	HR (95% CI)	*p* Value	HR (95% CI)	*p* Value
PFS	CD200 in Tumor	0.84 (0.59–1.21)	0.36	0.85 (0.58–1.24)	0.40
	CD200 in Stroma	0.91 (0.62–1.34)	0.61	0.83 (0.56–1.23)	0.34
OS	CD200 in Tumor	1.02 (0.71–1.47)	0.9	1.06 (0.70–1.60)	0.80
	CD200 in Stroma	1.01 (0.69–1.48)	0.97	1.08 (0.70–1.68)	0.72

## Data Availability

The data presented in this study are available on request from the corresponding author.

## Supplementary Materials

The following are available online at https://www.mdpi.com/article/10.3390/cancers13215501/s1, Figure S1: Block to block regressions of TIL and CAF markers in YTMA454, Figure S2: Block to block regressions of CD200 in YTMA454, Figure S3: Regressions of CD200 in different compartments and between CD200 and TIL and CAF markers in the stromal compartment in YTMA454, Figure S4: Overall survival of patients in YTMA454 based on expression of TIL and CAF biomarkers, Figure S5: Survival of patients in YTMA454 based on CD200 expression, Table S1: Univariate and multivariate analyses of overall survival of patients in YTMA454 based on expression of TIL and CAF biomarkers.
