# Supplementary material for: Multiplex Quantitative Analysis of Tumor-Infiltrating Lymphocytes, Cancer-Associated Fibroblasts, and CD200 in Pancreatic Cancer
Source: Cancers (Basel). 2021 Nov 2;13(21):5501. doi: 10.3390/cancers13215501 (PMC8583434; doi:10.3390/cancers13215501)
Supplement: Supplementary file 1 [file cancers-13-05501-s001.zip › cancers-1416830-supplementary.pdf]

Supplementary Files

9/28/2021

S1

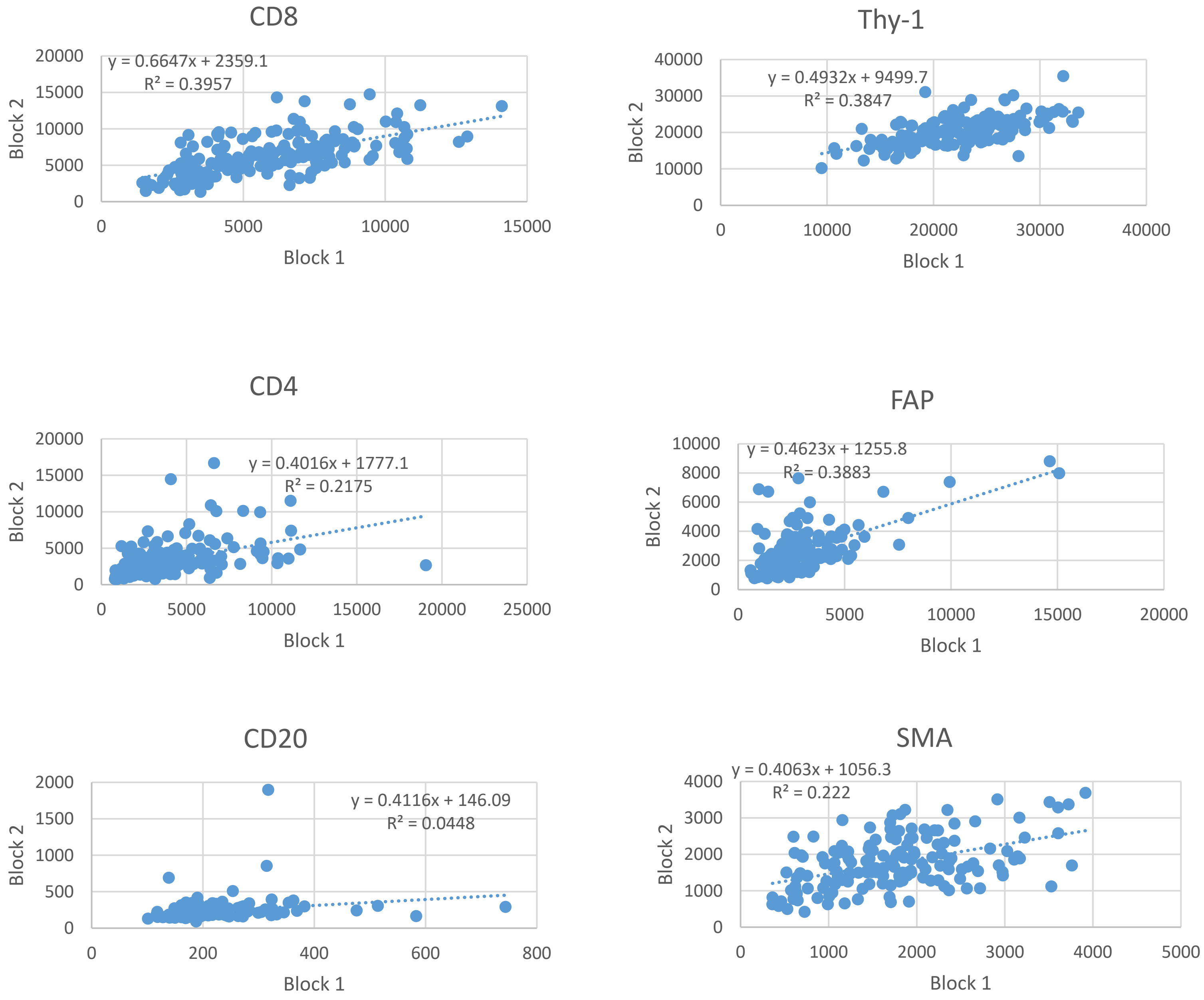
CD8
y = 0.6647x + 2359.1
R² = 0.3957
Block 2
Block 1
Thy-1
y = 0.4932x + 9499.7
R² = 0.3847
Block 2
Block 1
CD4
y = 0.4016x + 1777.1
R² = 0.2175
Block 2
Block 1
FAP
y = 0.4623x + 1255.8
R² = 0.3883
Block 2
Block 1
CD20
y = 0.4116x + 146.09
R² = 0.0448
Block 2
Block 1
SMA
y = 0.4063x + 1056.3
R² = 0.222
Block 2
Block 1

S2

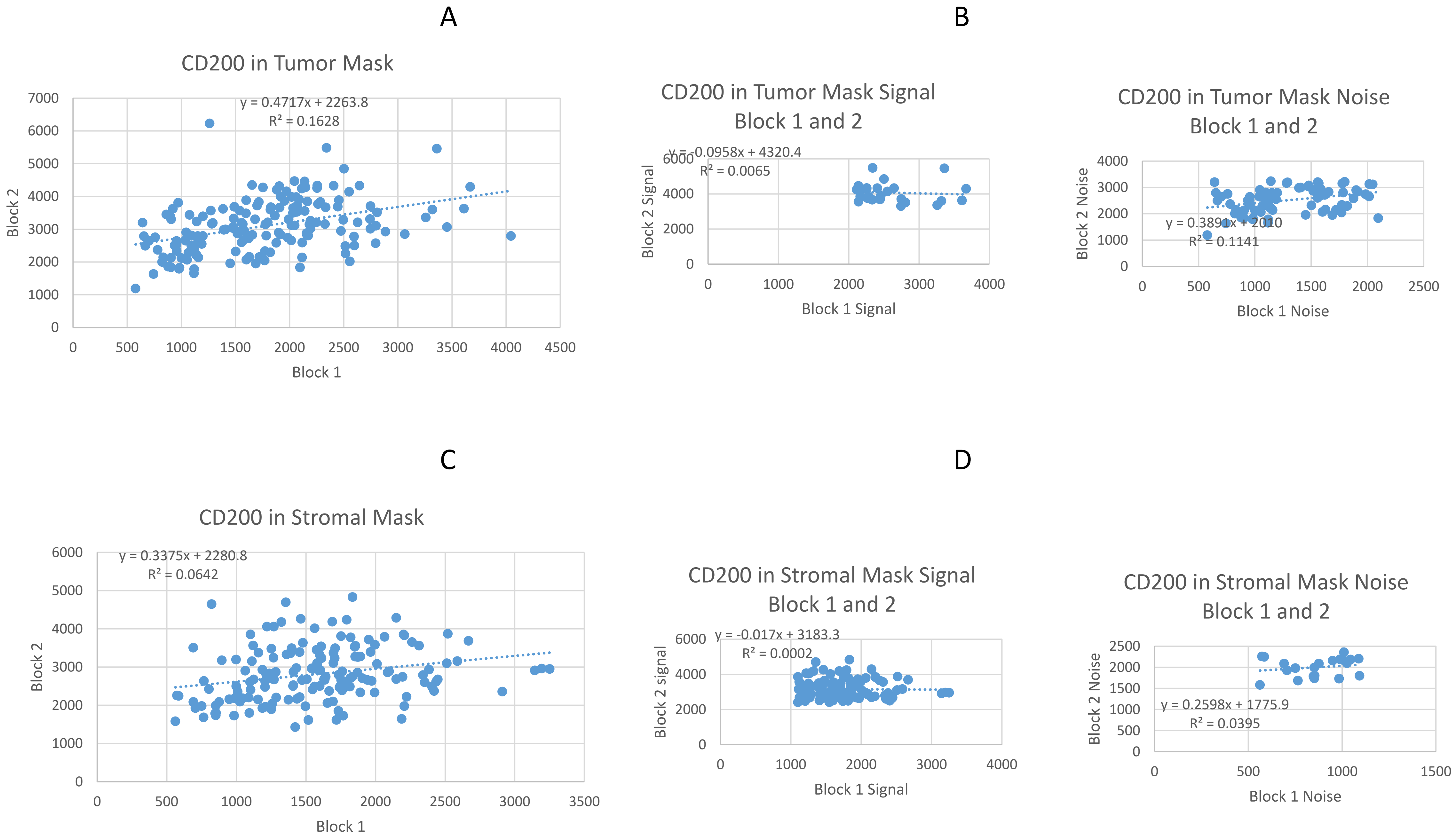
A
CD200 in Tumor Mask
y = 0.4717x + 2263.8
R² = 0.1628
Block 2
Block 1
B
CD200 in Tumor Mask Signal
Block 1 and 2
y = -0.0958x + 4320.4
R² = 0.0065
Block 2 Signal
Block 1 Signal
CD200 in Tumor Mask Noise
Block 1 and 2
y = 0.3891x + 2010
R² = 0.1141
Block 2 Noise
Block 1 Noise
C
CD200 in Stromal Mask
y = 0.3375x + 2280.8
R² = 0.0642
Block 2
Block 1
D
CD200 in Stromal Mask Signal
Block 1 and 2
y = -0.017x + 3183.3
R² = 0.0002
Block 2 signal
Block 1 Signal
CD200 in Stromal Mask Noise
Block 1 and 2
y = 0.2598x + 1775.9
R² = 0.0395
Block 2 Noise
Block 1 Noise

S3

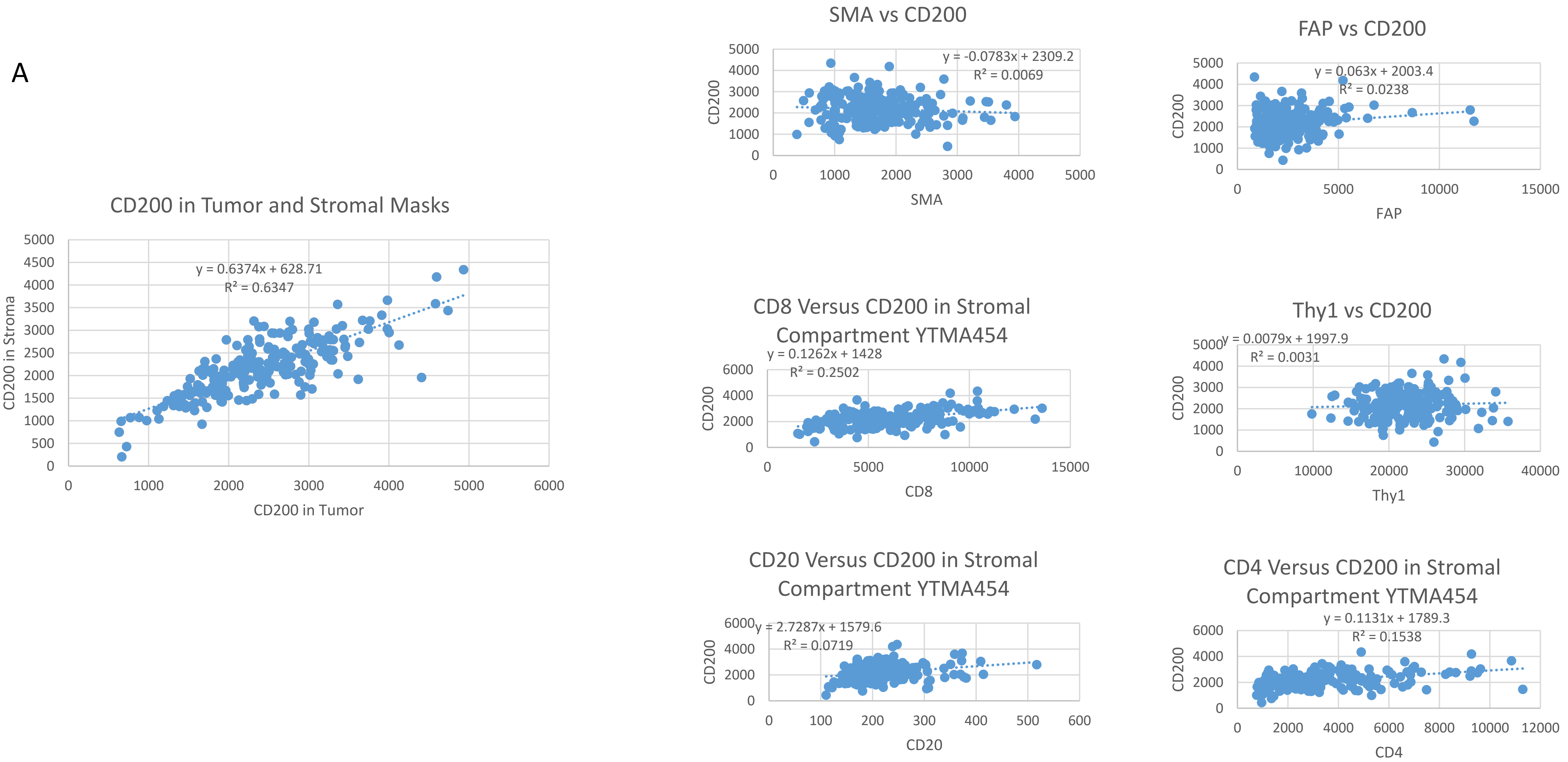
A
CD200 in Tumor and Stromal Masks
y = 0.6374x + 628.71
R² = 0.6347
CD200 in Stroma
CD200 in Tumor
0
500
1000
1500
2000
2500
3000
3500
4000
4500
5000
0
1000
2000
3000
4000
5000
6000
B
SMA vs CD200
y = -0.0783x + 2309.2
R² = 0.0069
CD200
SMA
0
1000
2000
3000
4000
5000
FAP vs CD200
y = 0.063x + 2003.4
R² = 0.0238
CD200
FAP
0
5000
10000
15000
CD8 Versus CD200 in Stromal Compartment YTMA454
y = 0.1262x + 1428
R² = 0.2502
CD200
CD8
0
2000
4000
6000
0
5000
10000
15000
Thy1 vs CD200
y = 0.0079x + 1997.9
R² = 0.0031
CD200
Thy1
0
10000
20000
30000
40000
CD20 Versus CD200 in Stromal Compartment YTMA454
y = 2.7287x + 1579.6
R² = 0.0719
CD200
CD20
0
100
200
300
400
500
600
CD4 Versus CD200 in Stromal Compartment YTMA454
y = 0.1131x + 1789.3
R² = 0.1538
CD200
CD4
0
2000
4000
6000
8000
10000
12000

S4

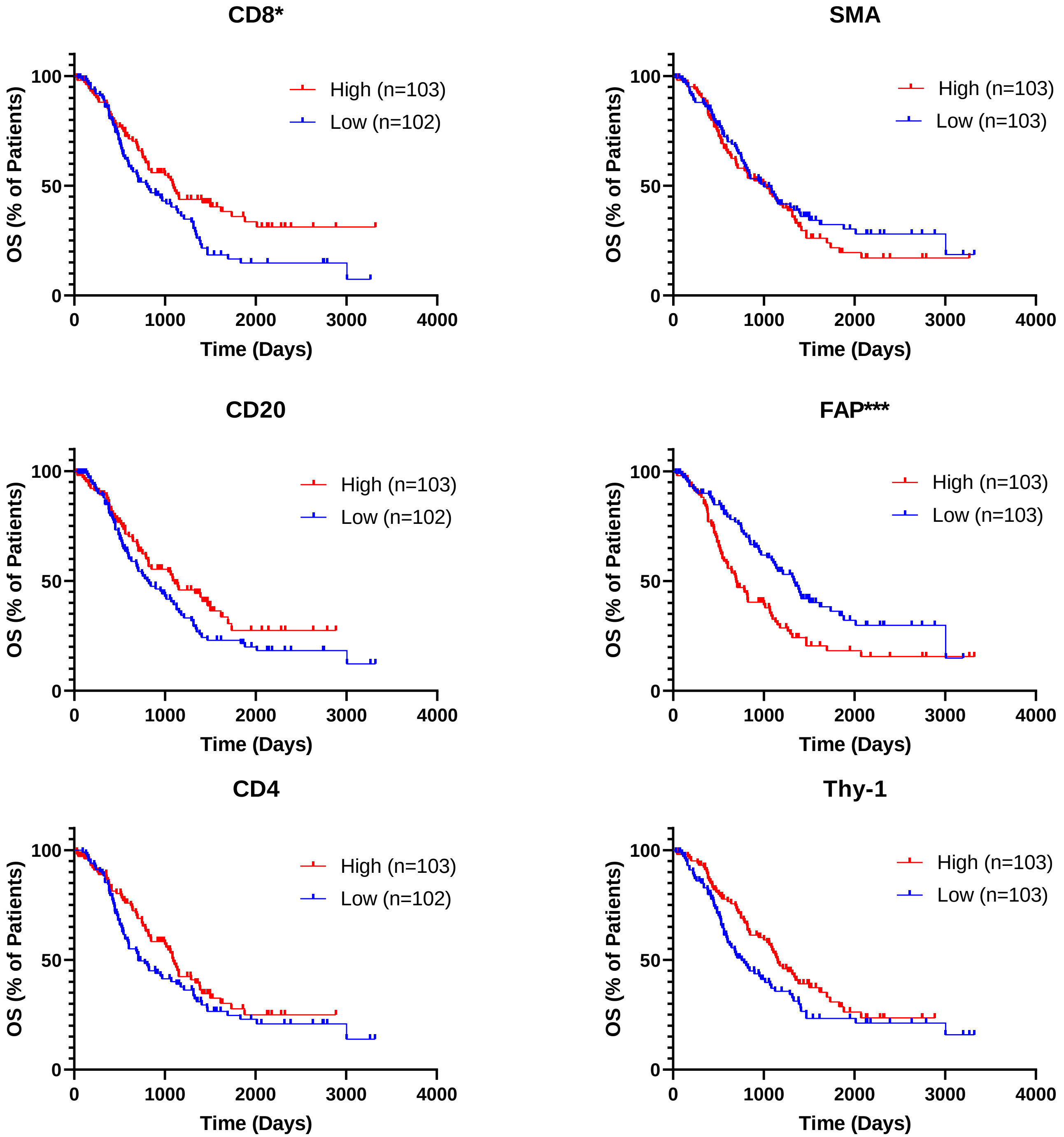
CD8*
High (n=103)
Low (n=102)
SMA
High (n=103)
Low (n=103)
CD20
High (n=103)
Low (n=102)
FAP***
High (n=103)
Low (n=103)
CD4
High (n=103)
Low (n=102)
Thy-1
High (n=103)
Low (n=103)
OS (% of Patients)
Time (Days)
0
50
100
1000
2000
3000
4000

Table S1

Protein	Univariable analysis		Multivariate analysis per variable		Multivariate analysis CD8 and FAP	
High/Low	HR (95% CI)	p value	HR (95% CI)	p value	HR (95% CI)	p value
CD4	0.78 (0.55-1.10)	0.16	0.82 (0.55-1.23)	0.34		
CD8	0.66 (0.47-0.93)	**0.016**	0.58 (0.38-0.87)	**0.0079**	0.64 (0.42-0.97)	**0.035**
CD20	0.74 (0.52-1.04)	0.081	0.71 (0.48-1.06)	0.097		
Thy-1	0.71 (0.51-1.01)	0.052	0.79 (0.52-1.18)	0.25		
FAP	1.80 (1.27-2.55)	**0.0007**	1.79 (1.20-2.68)	**0.0043**	1.63 (1.07-2.46)	**0.022**
SMA	1.18 (0.84-1.66)	0.34	1.18 (0.80-1.75)	0.4		

S5

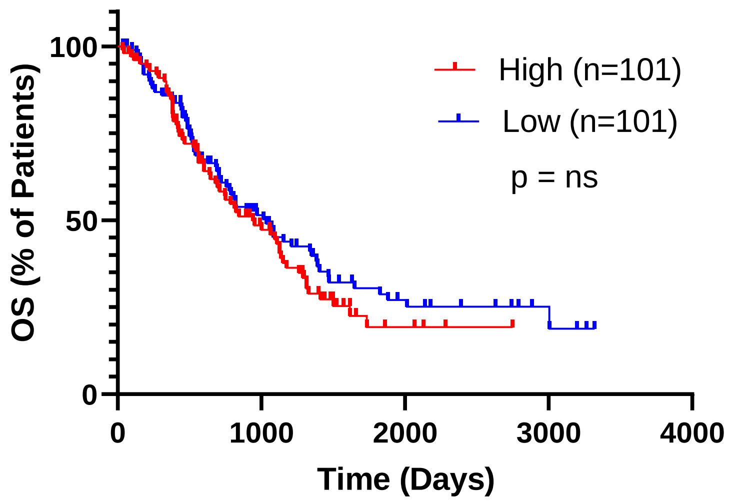

OS (% of Patients)
100
50
0
0
1000
2000
3000
4000
Time (Days)
High (n=101)
Low (n=101)
p = ns

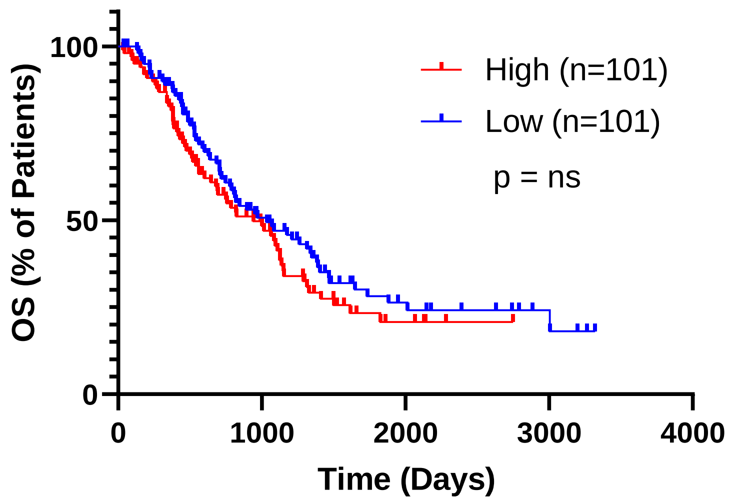

OS (% of Patients)
100
50
0
0
1000
2000
3000
4000
Time (Days)
High (n=101)
Low (n=101)
p = ns

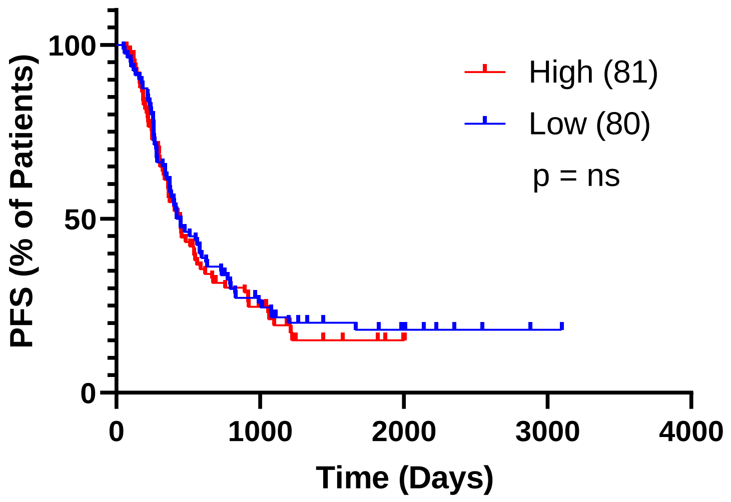

PFS (% of Patients)
100
50
0
0
1000
2000
3000
4000
Time (Days)
High (81)
Low (80)
p = ns

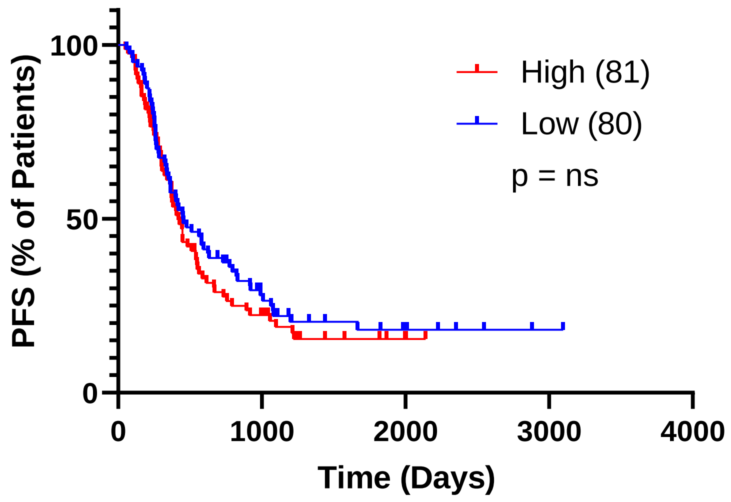

PFS (% of Patients)
100
50
0
0
1000
2000
3000
4000
Time (Days)
High (81)
Low (80)
p = ns
